# Mesenchymal Stem Cells and Selenium Nanoparticles Synergize with Low Dose of Gamma Radiation to Suppress Mammary Gland Carcinogenesis via Regulation of Tumor Microenvironment

**DOI:** 10.1007/s12011-022-03146-1

**Published:** 2022-02-09

**Authors:** Omayma A. R. Abozaid, Laila A. Rashed, Sawsan M. El-Sonbaty, Amira I. Abu-Elftouh, Esraa S. A. Ahmed

**Affiliations:** 1 Biochemistry Department, Faculty of Veterinary Medicine, Benha University, Banha, Egypt; 2 Medical Biochemistry and Molecular Biology Department, Faculty of Medicine, Cairo University, Cairo, Egypt; 3 Radiation Microbiology Department, National Center for Radiation Research and Technology, Egyptian Atomic Energy Authority, Cairo, Egypt; 4 Mansoura Regional Blood Transfusion Center, Mansoura, Egypt; 5 Radiation Biology Research Department, National Center for Radiation Research and Technology, Egyptian Atomic Energy Authority, Nasr City, Cairo, 11787 Egypt

**Keywords:** Mesenchymal stem cells, Selenium nanoparticles, Low-dose radiation, Breast cancer, Tumor suppressor/promoter genes, Tumor microenvironment

## Abstract

Breast cancer is one of the most prevalent and deadliest cancers among women in the world because of its aggressive behavior and inadequate response to conventional therapies. Mesenchymal stem cells (MSCs) combined with green nanomaterials could be an efficient tool in cell cancer therapy. This study examined the curative effects of bone marrow–derived mesenchymal stem cells (BM-MSCs) with selenium nanoparticles (SeNPs) coated with fermented soymilk and a low dose of gamma radiation (LDR) in DMBA-induced mammary gland carcinoma in female rats. DMBA-induced mammary gland carcinoma as marked by an elevation of mRNA level of cancer promoter genes (Serpin and MIF, LOX-1, and COL1A1) and serum level of VEGF, TNF-α, TGF-β, CA15-3, and caspase-3 with the reduction in mRNA level of suppressor gene (FST and ADRP). These deleterious effects were hampered after treatment with BM-MSCs (1 × 10^6^ cells/rat) once and daily administration of SeNPs (20 mg/kg body weight) and exposure once to (0.25 Gy) LDR. Finally, MSCs, SeNPs, and LDR notably modulated the expression of multiple tumor promoters and suppressor genes playing a role in breast cancer induction and suppression.

## Introduction

Breast cancer is one of the most common female malignant neoplasms of the mammary gland epithelial tissue. It is the 2^nd^ most prevalent malignancy worldwide and is the most prevalent cancer among Egyptian women [1]. It is the 2^nd^ type of cancer among the Egyptian population (16.4%) after liver cancer and is the first in the females (32.4%). Moreover, the mortality rate reaches 10.3% causing the death of more than nine thousand people in Egypt [2]. Although chemotherapy, radiotherapy, and surgery remain the mainstay treatment for breast cancer, these therapies are associated with severe adverse effects and patients can develop resistance to these agents [3]. Moreover, a lot of innovative approaches have recently been approved such as immunotherapy, conjugated antibodies and checkpoint inhibitors, and molecular-targeted therapy [4]. Even these modern anticancer modalities are associated with a number of often serious side effects [5]. Because of the increasing rates of morbidity and mortality of this disease and the desired patient-tailored therapy strategies, identification of new prognostic markers, therapeutic targets, and new therapeutic approaches is needed [6]. Furthermore, combined therapy has a better effect on continuous control of local tumors and the improvement of the cure rate compared with radiotherapy alone and chemotherapy or sequential therapy [7].

Low-dose radiation (LDR) gained attention in the field of radiotherapy [8]. Interestingly, Yang et al. [9] reported that LDR was more effective than conventional radiotherapy protocols as a potential control system during carcinogenesis without any side effects. In particular, LDR triggers an adaptive response via enhancing DNA repair, scavenging of free radicals, intercellular induction of apoptosis, and autocrine self-destruction and stimulating immune responses [10]. In addition to activation of many anticancer pathways such as secretion of various growth factors and cytokines and triggering natural killer cells [11, 12], another important property of LDR is the protection of the normal cells from oxidative stress damaging effect through induction of cell resistance against oxidative stress [13]. Low total body irradiation before and after conventional surgery, radiotherapy, and chemotherapy might reduce the chance of tumor recurrence and metastasis. Additionally, this combination reduces the total radiation dose and simultaneously improves the treatment efficacy of cancer accompanied by upregulated host anticancer immunity [14].

Mesenchymal stem cells (MSCs) are easily accessible multipotent cells that can be isolated from various tissues such as the bone marrow, adipose tissue, and umbilical cord blood. They have the potential to differentiate into multiple lineages [15]. They have important features that made them the most suitable choices for cell-based therapy for cancers. MSCs can preferentially be home to injured sites and tumor tissues as well as transactions with various cells in the tumor microenvironment in addition to the secretion of various trophic factors. These cells are easily accessible with low immunological responses, have the potential to differentiate, and are simply manipulated without the need for ethical concern. Moreover, MSCs can ameliorate the side effects of conventional anticancer therapies [16, 17]. Furthermore, He et al. [18] showed that a combination of mesenchymal stem cells with radiotherapy in the treatment of breast cancer can overcome the limited curative effect and enhance the radiosensitivity of cancer cells. Other studies showed that MSCs enhanced the radiotherapy effect on cancers likely through inhibition of tumor cell proliferation and enhancement of cancer cell apoptosis [19].

Nanotechnology has presented advantages compared to current chemotherapy] and has greatly improved the diagnosis and treatment of tumors [20]. Selenium nanoparticles (SeNPs) are promising therapeutic agents, due to their good bioavailability, higher biological activity, and low toxicity compared with inorganic and organic Se compounds [21]. SeNPs showed an attractive anticancer effect in various cancers such as liver cancer, breast cancer, prostate cancer, colon cancer, and lung cancer via induction of apoptosis as well as inhibition of proliferation, invasion, and metastasis. Moreover, SeNPs may act as a radiosensitizer and lower the side effects of radiotherapy [22]. Additionally, it was found that the combination of nanoparticles with chemotherapeutic agents overcomes cancer multidrug resistance and systemic toxicity and enhances the efficacy and cellular internalization of NPs [23]. Conjugation or surface modification of SeNPs was used to overcome the reduced cellular intake and enhance its anticancer adequacy by antibiotics, biomolecules, or phytochemical compounds present in microbes or plants [24]. Soymilk is rich in active phytochemical compounds as flavonoids (genistein), stilbenes (resveratrol), polyphenols (curcumin), and isothiocyanates, all have been shown to induce the apoptotic pathway in cancer cells preferentially over normal cells. Phytoestrogens may inhibit cancers in the breast, prostate, endometrial, thyroid, skin, and colon [25].

Accordingly, the present study aims to evaluate the mechanisms by which combined therapy using nano-Se, regenerative stem cell therapy, and radiotherapy attenuate mammary gland carcinoma microenvironment.

## Materials and Methods

### Preparation and Characterization of Selenium Nanoparticles (SeNPs)

#### Preparation of Fermented Soymilk

Organic soybean and distilled water (1:10 ratio) were mixed, heated to 100 °C for 30 min, and then filtered to obtain the soymilk. Fermented soymilk (FSM) was prepared according to the method of Chung et al. [26]. The microorganisms used in the fermenting process included *Lactobacillus acidophilus*, *Lactobacillus bulgaricus*, *Streptococcus lactis*, *Bifidobacteria*, and *Saccharomyces cerevisiae*. The broth was prepared as TGY medium (tryptone 5.0 g, yeast extract 5.0 g, glucose 1.0 g, distilled water 1.0 l, and pH 7.0) and Sabouraud dextrose media for yeast (glucose 40 g, peptone 10 g, distilled water 1 l, pH 5.6). The final fermented soymilk was heat-sterilized and filtered.

#### Selenium Nanoparticle Preparation

The aqueous part of fermented soy obtained was used as a precursor for the synthesis of SeNPs. The aqueous part of fermented soy (2 ml) was added dropwise into the 20-ml solution of SeO_2_ (10 mM), with vigorous stirring. The mixture was incubated by placing the solution onto a rotatory orbital shaker operating at 5 × *g*, 30 °C for 72 h in dark conditions. The reduction of selenium ions was monitored by measurement of absorption maximum wavelength 350 to 700 nm using a UV–Vis spectrophotometer [27].

#### Characterization of Selenium Nanoparticles

The determination of nanoparticular size and concentration is important for the biomedical use of nanoparticles. By transmission electron microscopy (TEM), the shape and size of SeNPs were determined from TEM (JEOL; model JEM2100, Japan) micrographs. Sample of SeNPs was analyzed through dynamic light scattering (DLS) by fluctuation in the intensity of scattered light produced by particles in Brownian motion using the Zetasizer [28]. Sample of SeNPs was analyzed for their functional groups presented on the nanoparticles, using the VERTEX 70 FTIR spectrometer, BRUKER. The samples were scanned in the range of 400–4000 wavenumber cm^−1^ and results are represented in % transmittance. For ultraviolet–visible (UV/Vis) absorption spectroscopy, the SeNPs sample was scanned using the Shimadzu 1,700 UV–Vis spectrophotometer.

### Isolation and Propagation of BM-Derived MSCs from Rats

Bone marrow was harvested by flushing the tibiae and femurs of 6-week-old male rats with Dulbecco’s modified Eagle’s medium (DMEM, GIBCO/BRL) supplemented with 10% fetal bovine serum (GIBCO/BRL). Nucleated cells were isolated with a density gradient [Ficoll/Paque (Pharmacia)] and resuspended in a complete culture medium supplemented with 1% penicillin and streptomycin (GIBCO/BRL). Cells were incubated at 37 °C in 5% humidified CO_2_ for 12–14 days as primary culture. The medium was changed every 2–3 days [29].

### Identification of BM-Derived MSCs

BM-derived MSCs were identified as being MSCs by their morphology, adherence, and detection of CD90, CD105, and CD34 which are the surface markers of rat mesenchymal stem cells that were identified by a flow cytometer.

### Labeling of Stem Cells with PKH26

In the current work, undifferentiated MSCs were labeled with PKH26 according to the manufacturer’s recommendations (Sigma, St. Louis, MO, USA). Cells were injected intraperitoneally into the rat. After 1 month, mammary gland tissue was examined with a fluorescence microscope to detect the cells stained with PKH26.

### *Determination of Acute Toxicity (LD*_*50*_*) of SeNPs in Rats*

An initial step in the evaluation of the toxic characteristics of a substance is the determination of LD_50_ (the dose causing death to 50% of the tested group of animals) [30]. Fifty virgin female Swiss albino rats weighing 100–120 g were grouped into 5 and orally administrated with SeNPs in ascending doses (12.5, 25, 50, and 100 mg/kg b.w). Rats were observed for toxicity signs and mortality. It was found that 20 mg/kg is safe and it was administered to rats.

LD_50_ was calculated according to the following formula:

$${\mathrm{LD}}_{50} = Dm - [\sum (a \times b) / N]$$

where

*Dm* is the apparent least lethal dose to all animals in a group;

*a* is the dose difference;

*b* is the mean mortality;

*N* is the number of animals in each group (6 rats);

 and *∑* is the sum of (*a* × *b*).

### Preparation of 7,12-Dimethylbenz(a)anthracene (DMBA)

The 7,12-dimethylbenz(a)anthracene (DMBA) from Sigma Co., USA, was freshly prepared by dissolving in sesame oil and administered at a dose of 50 mg/kg body weight intraperitoneal (i.p.) along the ventral midline of the animal, halfway between the third and fourth pairs of mammary glands [31].

### Radiation Facility

Rats’ whole bodies were irradiated in the morning at 10 ± 15 a.m. The animals were exposed to 0.25 Gy γ-radiation delivered once at a dose rate of 0.423 Gy/min according to Frey et al. [32] which was calculated according to the Dosimetry Department guidelines at the National Center for Radiation Research and Technology (NCRRT) (Nasr City, Cairo, Egypt). Irradiation of rats was carried out using a Canadian Gamma Cell-40 (137Cs) at NCRRT.

### Experimental Animals

Virgin female Swiss albino rats of 4–5 weeks’ age, with a body weight range of 80–100 g were purchased from the animal breeding house of The Nile Company for Pharmaceutical Drugs (Cairo, Egypt). Throughout the experimental period, rats were allowed ad libitum access to food and water and housed under the same laboratory conditions with a light/dark cycle of 12 h, humidity of 50 ± 15%, and temperature of 22 ± 2 °C. The study was approved by the Ethics Committee of the National Center for Radiation Research and Technology, followed by the 3Rs principles for animal experimentation, and operated according to the CIOMS and ICLAS International Guiding Principles for Biomedical Research Involving Animals, 2012.

### Experimental Groups

**Fig. 1:**
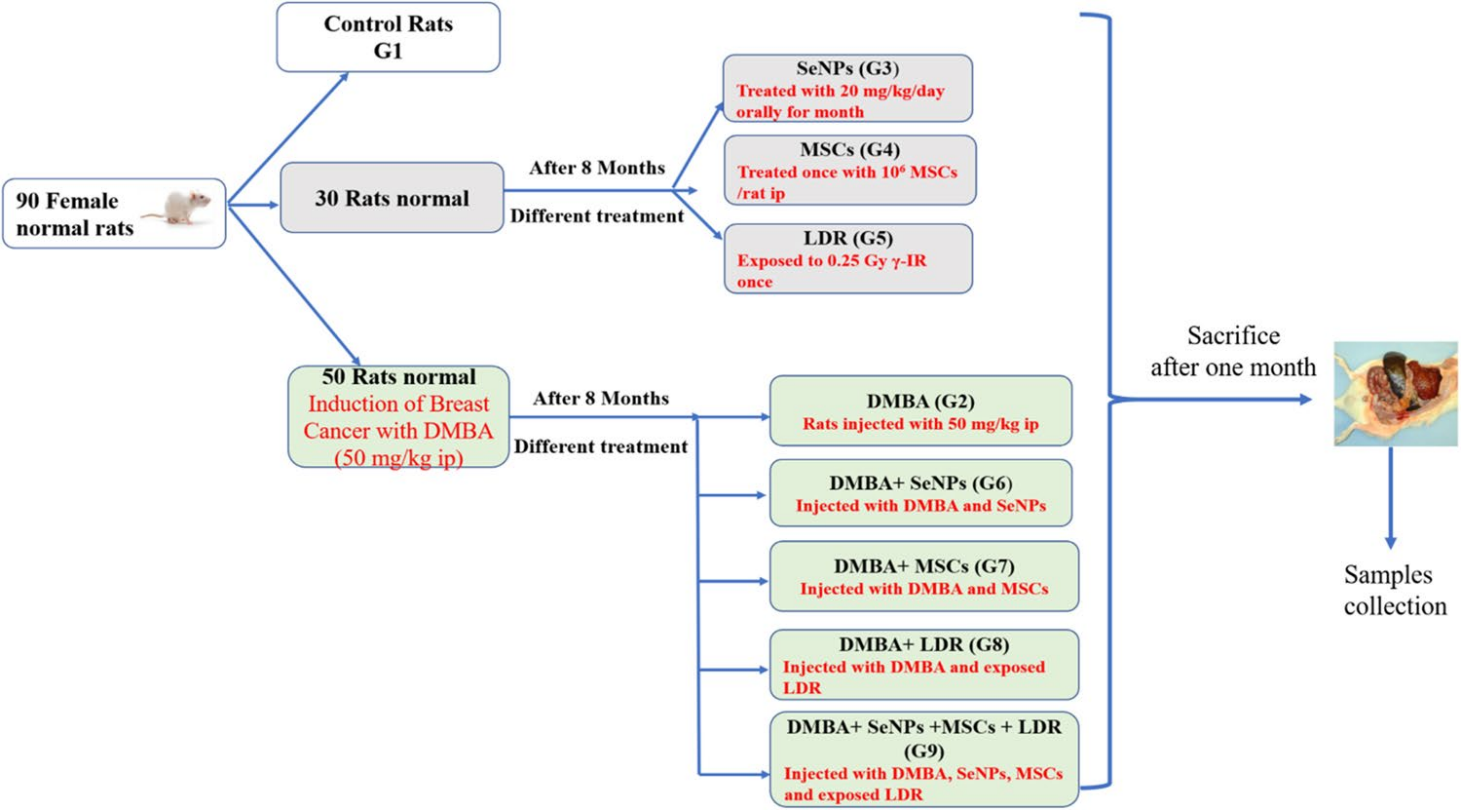
Experimental design and treatment protocol for different groups. DMBA, 7,12-dimethylbenz(a)anthracene; SeNPs, selenium nanoparticles; LDR, low-dose radiation; MSCs, mesenchymal stem cells

After 1 week of acclimatization, the 90 virgin female rats have equally divided into 9 groups, ten rats each, and the experimental design is illustrated in Fig. 1 as follows:

- Group (1) Control: healthy female rats received saline orally by gavage.
- Group (2) DMBA: set as the mammary gland carcinoma model in which rats were injected with DMBA intraperitoneally at a dose of 50 mg/kg once during 8 months.
- Group (3) SeNPs: rats were kept as the control group and after 8 months, they were administrated by gavage with SeNPs (20 mg/kg body weight) daily for 1 month.
- Group (4) MSCs: rats were kept as the control group and after 8 months, they were injected i.p. with MSCs 10^6^/rat once.
- Group (5) LDR: rats were kept as the control group and after 8 months, they were exposed to a single dose (0.25 Gy) of whole-body γ-radiation.
- Group (6) DMBA + SeNPs: rats were injected with DMBA as group (2); then, they were treated with SeNPs similar to group (3).
- Group (7) DMBA + MSCs: rats were injected with DMBA as group (2); then, they were treated with MSC similar to group (4).
- Group (8) DMBA + LDRR: rats were injected with DMBA as group (2); then, they were exposed to γ-radiation as group (5).
- Group (9) DMBA + MSCs + SeNPs + LDR: rats were injected with DMBA as group (2); then, they were exposed to LDR as group (5) and treated with SeNPs as group (3), after that they were treated with MSC as group (4).

At the end of the treatment period, animals were anesthetized using urethane, and blood was drawn from the vena cava. Mammary gland tissue was rapidly excised and divided into two parts. One portion was used for the histopathological examination and kept in 10% formaldehyde, while the other part of the tissue was homogenized for biochemical and molecular analyses.

### Biochemical Measurements Using ELISA

Serum from each group was assayed for cancer antigen 15–3 (CA15-3) using enzyme-linked immunosorbent assay (ELISA) kit, TGF-β level using rat TGF-β sandwich ELISA kit, TNF-α level using rat TNF-α sandwich ELISA kit, VEGF level using rat VEGF sandwich ELISA kit, and caspase-3 (Casp-3) level using rat caspase-3 ELISA kit (all were purchased from MyBioSource, Inc. USA).

### Quantitative Real-Time Polymerase Chain Reaction

Total mRNA was isolated using a QIAGEN tissue extraction kit (QIAGEN, USA) according to the instructions of the manufacturer. Quantitative real-time polymerase chain reaction (qRT-PCR) amplification and analysis were performed using Applied Biosystems with software version 3.1 (StepOne™, USA). The qRT-PCR assay with the primer (Table 1) sets was optimized at the annealing temperature. All complementary DNAs (cDNAs) were in duplicate and included previously prepared samples for SerpinE, MIF, ADRP, FST, LOXL-1, and COL1A1 with beta-actin (β-actin) as an internal control, and water is used as a non-template control to confirm the absence of DNA contamination in the reaction mixture.

**Table 1:** List of primer sequence

Gene	Accession no.	Primer sequence
ADRP	XM_008763778.1	F: 5′- CTT GTG TCC TCC GCT TAT GTC AGT -3′
		R: 5′- CTG CTC CTT TGG TCT TAT CCA CCA -3′
FST	XM_006231954.3	F: 5′- TGCTGCTACTCTGCCAATTC -3′
		R: 5′- TGCAACACTCTTCCTTGCTC -3′
Serpin1	NM_012620.1	F: 5′- GACACGCCATAGGGAGAGAAG -3′
		R: 5′- TCTGGGAAAGGGTTCACTTTACC -3′
MIF	NM_001111330	F: 5′- TGCCCAGAACCGCAACTACAGTAA -3′
		R: 5′- TCGCTACCGGTGGATAAACACAGA -3′
LOX-1	NC_005117.4	F: 5′- AGATCCAGACTGTGAAGGACCAGC -3′
		R: 5′- CAGGCACCACCATGGAGAGTAAAG -3′
COL1A1	NM_053304.1	F: 5′- ATCAGCCCAAACCCCAAGGAGA -3′
		R: 5′- CGCAGGAAGGTCAGCTGGATAG -3′
β-Actin	XM_017587861.1	F: 5′ -TGTTGTCCCTGTATGCCTCT -3′
		R: 3′ -TAATGTCACGCACGATTTCC -5′

### Histopathological Examination

Mammary gland tissues were cut into suitable pieces and fixed in neutral buffered formalin (10%) for 24 h, according to the method adopted by Banchroft et al. [33] Tissue sections were examined using a light microscope for histopathological investigation.

### Statistical Analysis

All data are presented as the mean ± standard error of the mean (SEM). Using the statistical package SPSS (Statistical Package for the Social Sciences) version 20, the comparisons between groups were performed using one-way analysis of variance (ANOVA) followed by LSD. The difference between means is considered statistically significant at *P* < 0.05.

## Results

### Characterization of SeNPs

**Fig. 2:**
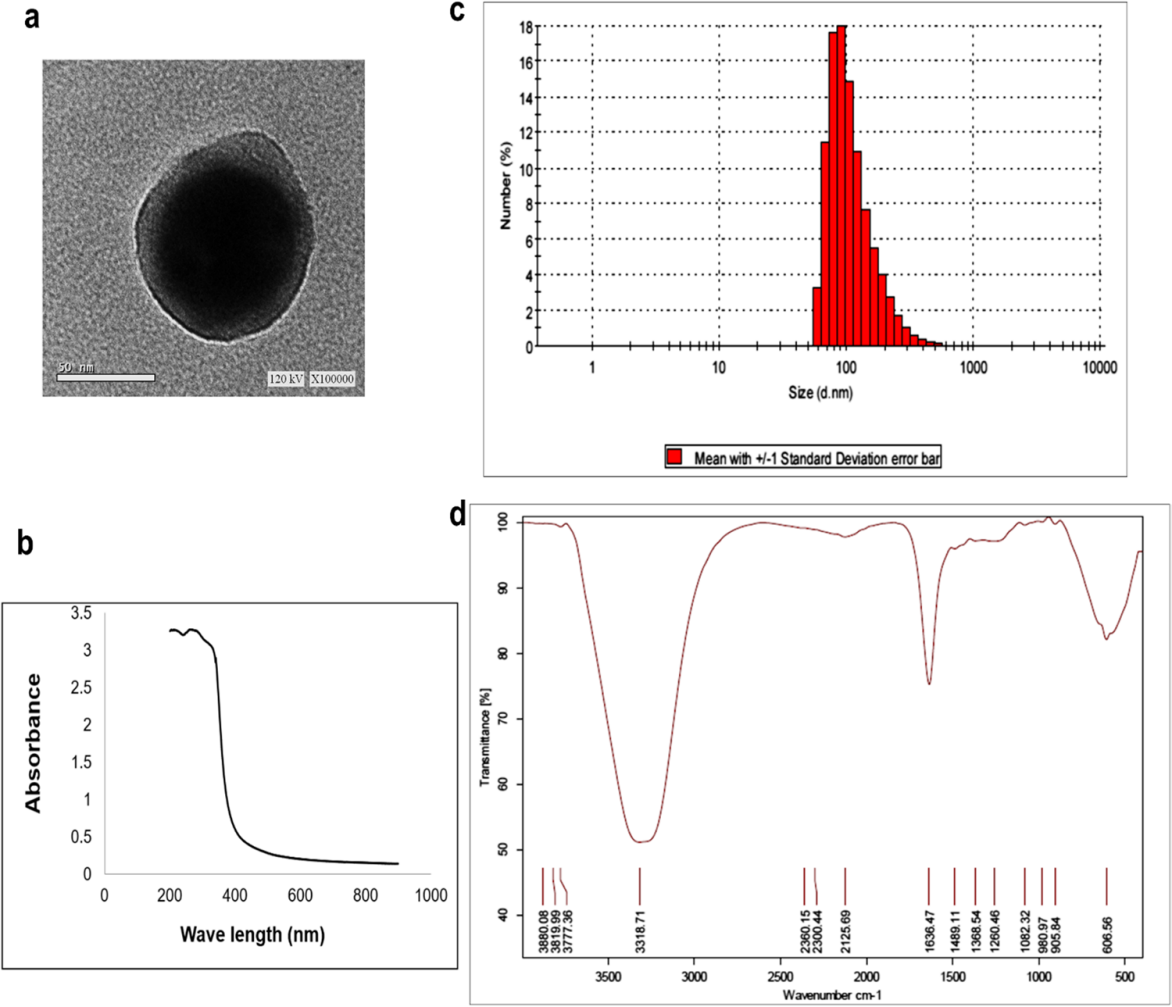
Characterization of SeNPs. (**a**) Transmission electron microscope photo of selenium nanoparticles. (**b**) Dynamic light scattering. (**c**) UV–Vis spectrum. (**d**) Fourier transform infrared spectroscopy

As illustrated in Fig. 2a, the results of TEM showed that the selenium nanoparticles are of the spherical shape of different sizes ranging from 12- to 17-nm properties. UV–Vis spectroscopic analysis of SeNPs showed a surface plasmon absorption band with a maximum absorbance at 262.5 nm which can be taken as an indication for SeNP formation (Fig. 2b). DLS studies revealed that the mean size of the nanoparticles ranges from 58.77 up to 531.2 nm with a high distribution of 91.28 nm reaching 18% as shown in Fig. 2c. The FTIR of SeNPs coated with fermented soy showed strong peaks at 3318.10 cm^−1^, 1636.93 cm^−1^, and 602.12 cm^−1^ which represents the hydroxyl group, amide I bonds of proteins, and a single bond of C–H or C–N respectively indicating the interactions of nanoparticles with proteins and phytochemicals of fermented soymilk. Overall, it can be concluded that the proteins in fermented soy extract are adsorbed as a layer over the synthesized SeNPs, thereby stabilizing the nanoparticles formed through the surface-bound proteins (Fig. 2d).

### Identification of BM-Derived MSCs from Rat

**Fig. 3:**
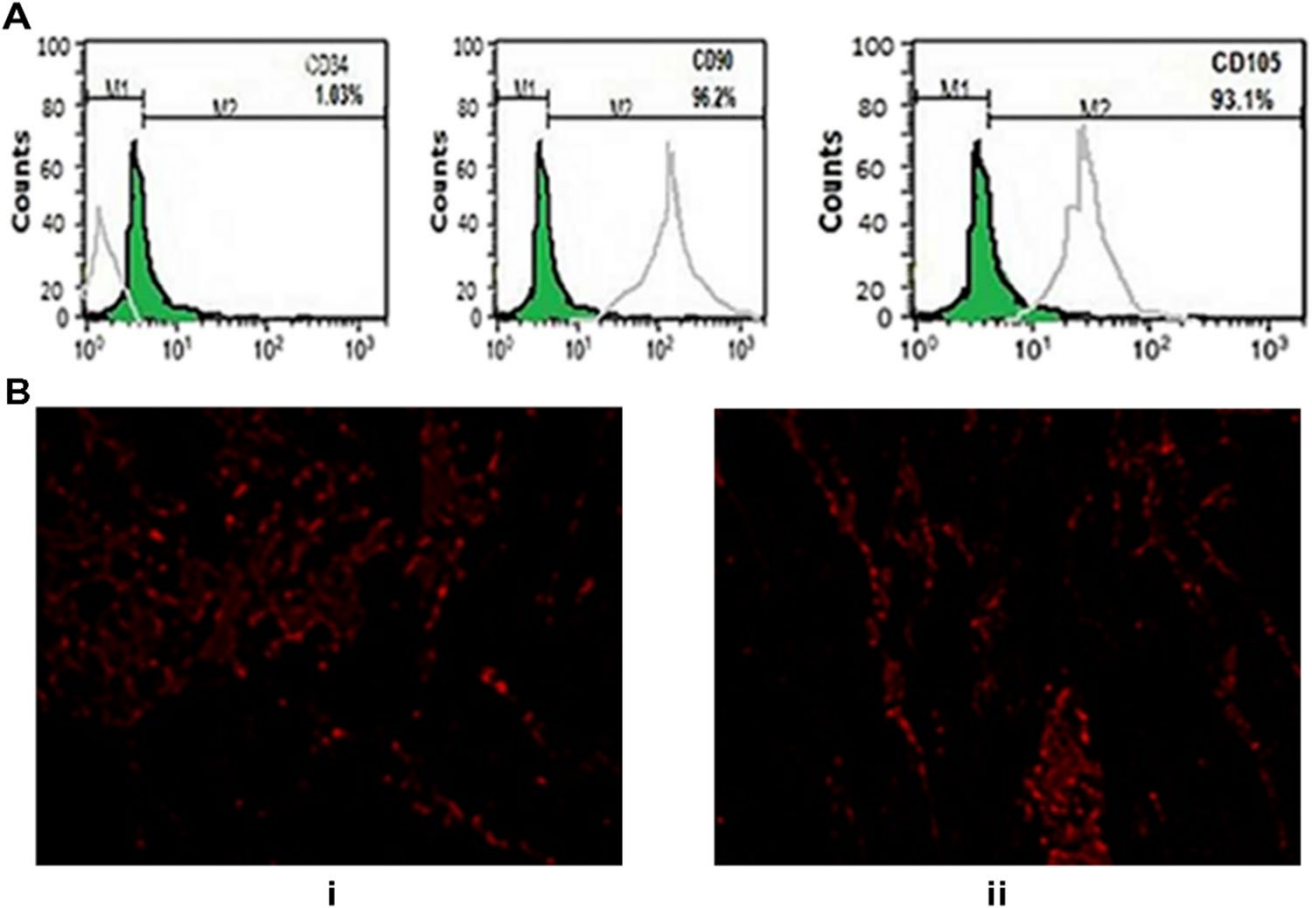
(**A**) Immunophenotypic characterization of BM-MSCs by flow cytometry. (**B**) Fluorescence microscope photos of BM-MSCs labeled by PKH26 engraftment in the breast tissue in DMBA + MSCs (**B, i**) and DMBA + MSCs + SeNPs + LDR (**B, ii**), respectively

The immunophenotype of BM-MSCs was examined by flow cytometry. As shown in Fig. 3A, BM-MSCs were negative for the hematopoietic marker (CD34), while strongly positive for mesenchymal stem cell–specific markers including CD90 and CD105. The gray histograms represent antibody labeled cells while the green histogram shows the profile of the isotype control.

#### Tracking of Injected Labeled MSCs

The BM-MSCs were labeled by PKH26 to track their engraftment in the breast tissue. The PKH26-labelled injected MSCs engrafted in the breast tissue of both groups DMBA + MSCs (Fig. 3B, i) and DMBA + MSCs + SeNPs + LDR (Fig. 3B, ii).

###  Effect of LDR, SeNPs, and MSCs on CA15-3 Level

**Fig. 4:**
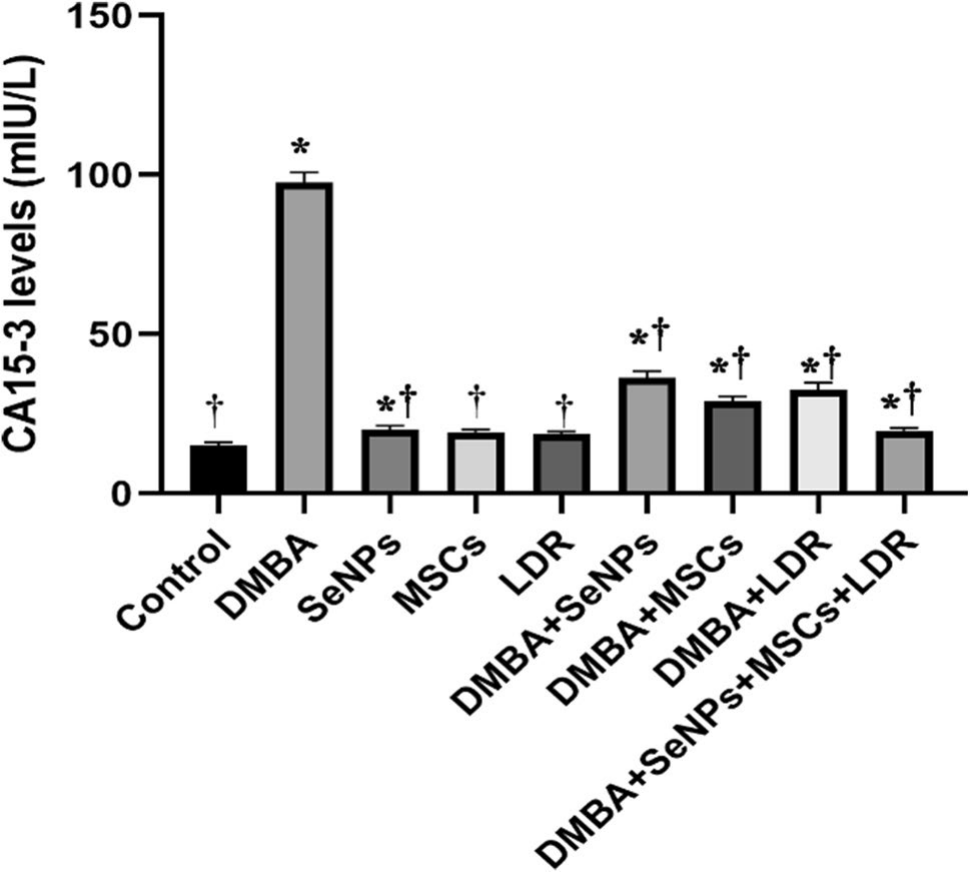
Effect of LDR, SeNPs, and MSCs on CA15-3 level. Data are presented as the means ± SE. ⁎, †, and ‡ denote significant change at *p* < 0.05 versus control and DMBA groups, respectively

Cancer antigen 15–3 (CA15-3) is an important tumor marker in breast cancer. The obtained results revealed a significantly elevated level of CA15-3 in the mammary gland tissue of the DMBA group compared to the control group. While, upon treatment with LDR, SeNPs, and MSCs, either alone or combined, the levels of CA15-3 were effectively reduced as shown in Fig. 4.

### The Potential Effect of LDR, SeNPs, and MSCs on Tumor Suppressor Genes (ADRP and FST)

**Fig. 5:**
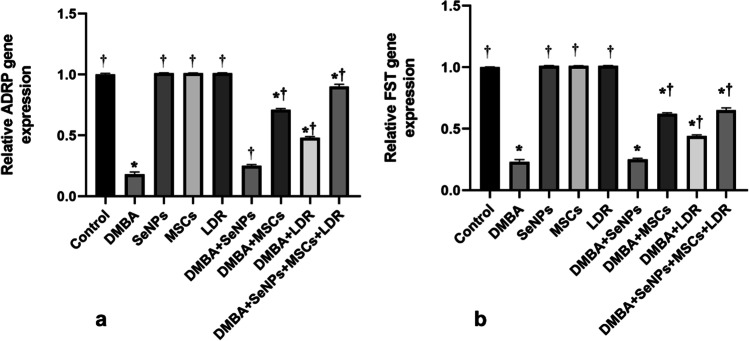
Effect of LDR, SeNPs, and MSCs on tumor suppressor genes ADRP (**a**) and FST (**b**). Data are presented as the means ± SE. ⁎, †, and ‡ denote significant change at *p* < 0.05 versus control and DMBA groups, respectively

Results in Fig. 5a, b illustrated that mRNA gene expressions of the ADRP and FST were significantly downregulated in the DMBA group compared to the control group. However, treatment with low-dose gamma IR (LDR), SeNPs, and MSCs either alone or combined markedly upregulated the gene expression of both ADRP and FST. This may suggest the decreased invasiveness of breast cancer with the treatment.

### Effect of LDR, SeNPs, and MSC Treatment on Expression of Genes Involved in Cancer Metastasis and Angiogenesis (LOX-1, COL1A1, Serpin, and VEGF)

**Fig. 6:**
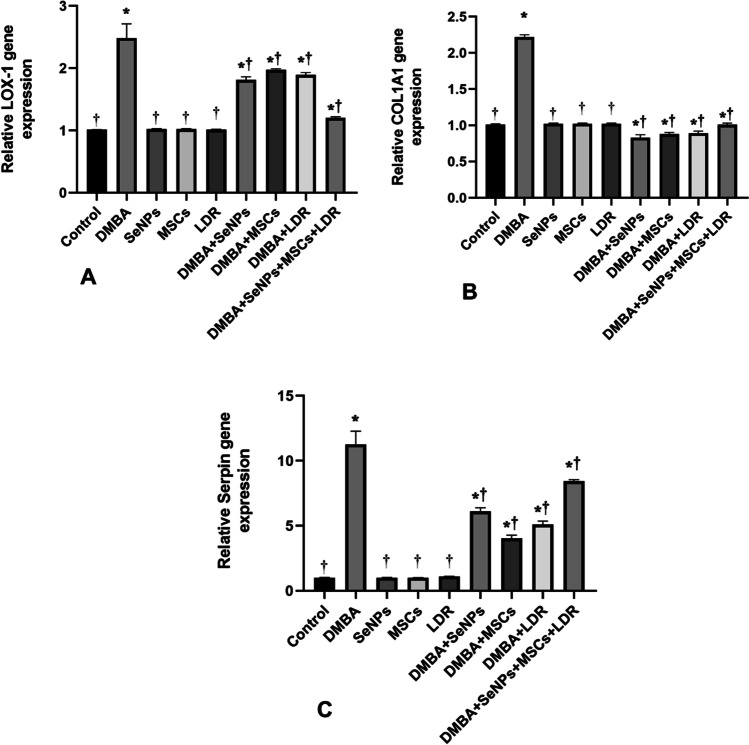
Effect of LDR, SeNPs, and MSCs on the expression of tumor promoter genes LOX-1 (**A**), COL1A1 (**B**), and SerpinE2 (**C**). Data are presented as the means ± SE. ⁎, †, and ‡ denote significant change at *p* < 0.05 versus control and DMBA groups, respectively

**Fig. 7:**
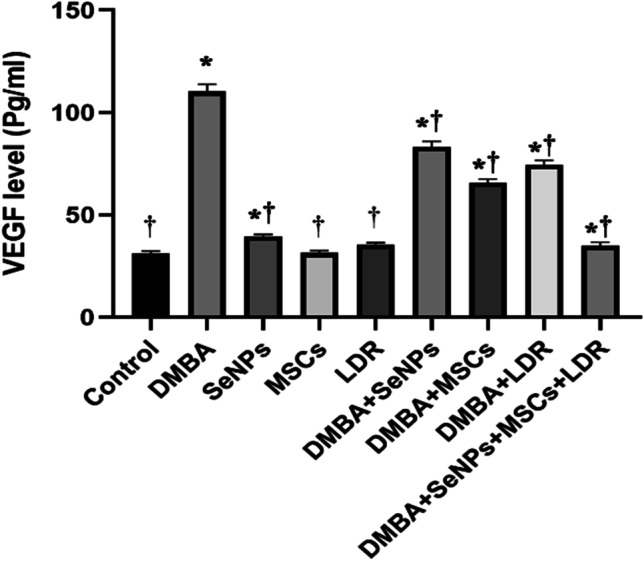
Effect of LDR, SeNPs, and MSCs on the level of VEGF. Data are presented as the means ± SE. ⁎, †, and ‡ denote significant change at *p* < 0.05 versus control and DMBA groups, respectively

The mRNA gene expressions of the LOX-1, Serpin, and COL1A1 were significantly upregulated together with a higher level of VEGF in the DMBA group compared to the control group as illustrated in Figs. 6A–C and 7. Treatment with LDR, SeNPs, and MSCs alone or combined caused a notable downregulation in these gene expressions with a concomitant decline in the level of VEGF. Moreover, the combined effect of LDR, SeNPs, and MSCs has a slight decrement in the gene expression of the Serpin that was observed. These results may suggest the efficiency of different treatments against metastasis and angiogenesis of breast cancer.

### Effect of LDR, SeNPs, and MSCs on Inflammatory Markers Associated with Mammary Gland Carcinoma (MIF, TNF-α, and TGF-β)

**Fig. 8:**
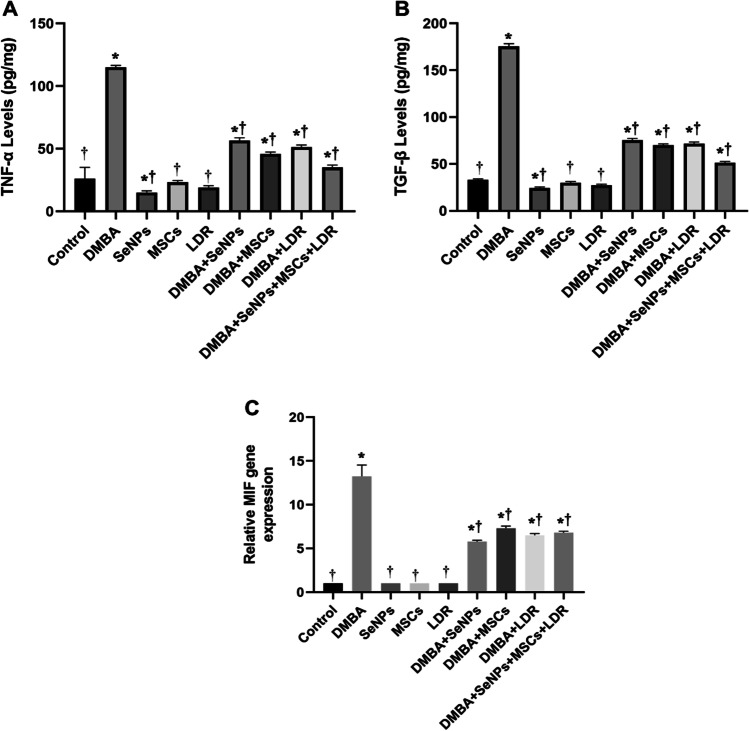
Effect of LDR, SeNPs, and MSCs on the levels of TNF-α (**A**), TGF-β (**B**), and MIF expression (**C**). Data are presented as the means ± SE. ⁎, †, and ‡ denote significant change at *p* < 0.05 versus control and DMBA groups, respectively

Due to the important role of inflammation in tumorigenesis, the levels of the inflammatory markers TNF-α and TGF-β as well as the mRNA expression of MIF were determined. Macrophage migration inhibitory factor (MIF) is an inflammatory molecule that is involved in a variety of neoplastic diseases. Moreover, TGF-β1 is involved in cell growth, differentiation, and inflammatory pathway. The obtained results revealed significantly elevated levels of TNF-α and TGF-β coupled with higher expression of the MIF mRNA in the DMBA group as compared to the control group confirming the tumor aggressiveness. In contrast, treatment with LDR, SeNPs, and MSCs either alone or combined remarkably reduced the levels of TNF-α and TGF-β and the mRNA expression of MIF (Fig. 8A–C).

### Effect of Treatment with LDR, SeNPs, and MSCs on Apoptosis (Caspase-3)

**Fig. 9:**
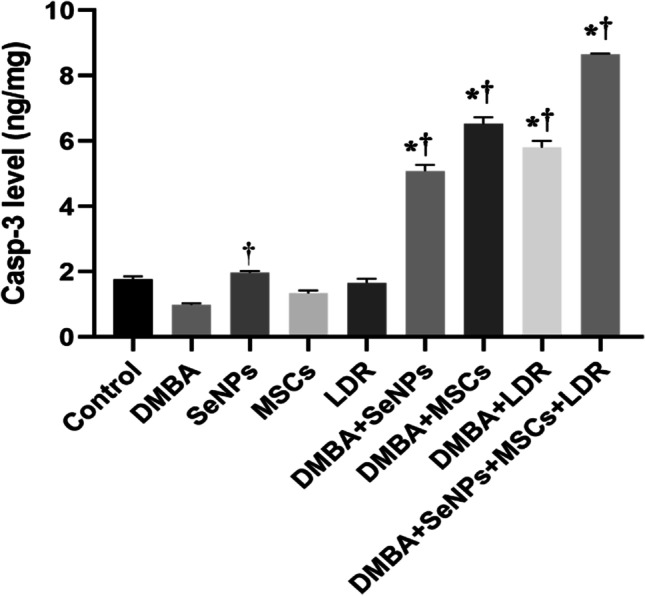
Effect of LDR, SeNPs, and MSCs on caspase-3 (Casp-3) levels. Data are presented as the means ± SE. ⁎, †, and ‡ denote significant change at *p* < 0.05 versus control and DMBA groups, respectively

The caspase-3 level was evaluated in the mammary tissue to elucidate the apoptotic effect of LDR, SeNPs, and MSCs. The current results showed a significant reduction in the level of Casp-3 in the DMBA group compared to the control group, which confirms breast cancer growth and proliferation. Conversely, treatment with LDR, SeNPs, and MSCs either alone or combined stimulates apoptosis in the cancerous tissue via marked increment in the level of Casp-3. Therefore, the combined treatment of SeNPs, MSCs, and LDR acts synergistically and triggers apoptosis and tumor cell death (Fig. 9).

### Histopathological Findings

**Fig. 10:**
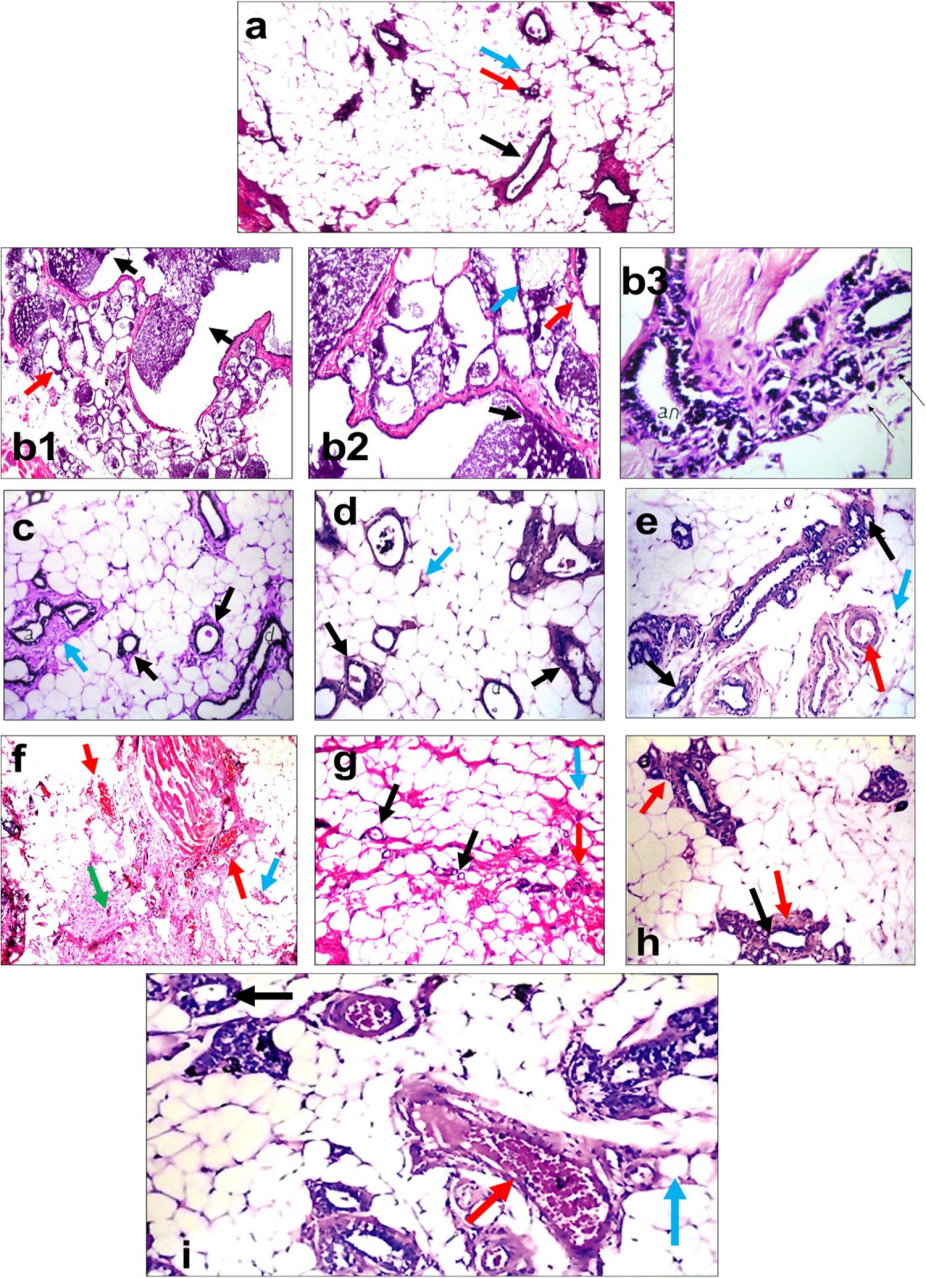
Effect of SeNPs, MSCs, and low dose of gamma radiation on mammary gland carcinogenesis of female rats. Sections in the mammary gland of the control group (**a**): showing average ducts with average epithelial lining (black arrows) embedded in average fibro-fatty stroma (red arrow) (H&E × 400). DMBA group (**b1**): mammary gland showing markedly dilated ducts with retained secretions (black arrows) and markedly necrotic fat cells (red arrow) (H&E × 200). Moreover, the high-power view (**b2**) showing markedly dilated ducts with retained secretions (black arrow) and scattered proliferating pleomorphic cells (red arrow), and markedly necrotic fat cells (blue arrow) (H&E × 400). Also, b3 showing infiltration of the stroma by pleomorphic neoplastic cells (black arrow), and ducts lined by a single layer of neoplastic cells (red arrow) (H&E × 400). SeNPs group (**c**) showing average ducts with average epithelial lining (black arrow) and average fibro-fatty stroma (blue arrow) (H&E × 200). MSCs group (**d**) showing average ducts with average epithelial lining (black arrows), average acini (red arrow), and average fibro-fatty stroma (blue arrow) (H&E × 200). Radiation group (**e**) showing ducts with average epithelial lining (black arrows) and dilated congested blood vessels (red arrow) with scattered peri-vascular inflammatory infiltrate (blue arrow) (H&E × 200). DMBA + SeNPs group (**f**) showing average ducts with stratified epithelial lining (black arrow), markedly dilated congested blood vessels (red arrows), average fibro-fatty stroma (blue arrow), and scattered inflammatory cells (green arrow) (H&E × 200). DMBA + MSCs group (**g**) showing small-sized ducts with flattened epithelial lining (black arrow) and excess stromal fibrous tissue with proliferating fibroblasts (red arrow) (H&E × 200). DMBA + radiation group (**h**) showing ducts with flattened epithelial lining (black arrow) and excess peri-ductal fibrous tissue (red arrow) (H&E × 200). DMBA + SeNPs + MSCs + radiation group (**i**) mammary gland showing ducts with average epithelial lining (black arrow), dilated congested blood vessels (red arrow), and average fibro-fatty stroma (blue arrow) (H&E × 200)

Figure 10a, c, d shows normal structure of mammary gland tissues consisting of average ducts, epithelial lining, and acini embedded in the average fibro-fatty stroma representing the control, SeNP, and MSC groups. While exposing rats to low-dose gamma radiation causes dilated congested blood vessels (Fig. 10, e), on the other hand, in the DMBA-induced mammary gland carcinoma, there are markedly dilated ducts with retained secretions, infiltration of the stroma by pleomorphic hyperchromatic neoplastic cells, ducts lined by a single layer of neoplastic cells, and markedly necrotic fat cells (Fig. 10b). With different treatment either alone or in combination, a gradual restoration of the normal architecture of mammary gland tissue was observed (Fig. 10f–i).

## Discussion

In this study, we have shown that the combined treatment with SeNPs, MSCs, and low dose of gamma radiation produced synergistic effects on breast cancer.

The effectiveness of using a combinational therapy including SeNPs, MSCs, and LDR was elucidated by measuring the expression of some genes including Serpin, MIF, LOX-1, COL1A1, FST, and ADRP that play a role in the tumor microenvironment. Besides that, the levels of inflammatory markers and VEGF were also detected. The obtained results revealed that injection of DMBA resulted in mammary gland carcinoma confirmed with the histopathological results and higher levels of the CA15-3. DMBA through oxidative stress displays reproducible chromosomal aberration and elevated expression of oncogenes and onco-pathways and eventually generates malignancies [34]. Breast cancer is associated with a dramatic downregulation of ADRP and FST along with upregulation of the Serpin, LOX-1, COL1A1, and MIF gene expressions, respectively. Therefore, apoptosis was suppressed by lowering the level of caspase-3. Angiogenesis is also involved via higher levels of VEGF.

The results coincide with those of Martinez et al. [35] and Kindt et al. [36] who confirmed the overexpression of MIF in breast cancer which indicates the crucial role in tumor progression. Moreover, Xu et al. [37] reported that the increased expression of MIF was associated with higher levels of VEGF confirming the angiogenic function of the MIF. As a whole, MIF plays an important role in carcinogenesis via promoting proliferation and migration and inhibiting autophagy and apoptosis. Besides that, it is a vigorous immunosuppressor and affects the tumor microenvironment leading to angiogenesis, migration, invasion, and metastases [38, 39].

Furthermore, various studies have shown that the dysregulation of the tumor microenvironments and its extracellular matrix (ECM) have critical roles in the survival, growth, and metastatic progression of cancer especially breast cancer [6]. SerpinE1 is a molecule involved in several human malignancies. It plays an important role in signal transduction, cell adhesion, and cell migration [40]. A high level of SerpinE1 has been revealed to be associated with a poor prognosis of breast cancer [41] where it was significantly associated with metastasis and invasion; consequently, it has been validated clinically in breast cancer as a biomarker [42]. Supporting this possibility, our results agree with Nabatchican et al. [43] and Vaillant et al. [44] indicating the overexpression of SerpinE2 in breast cancer tissues exhibiting the essential role of SerpinE2 in the progression and metastasis of malignant breast cancer.

Moreover, the invasion and aggression of breast cancer were accompanied by extracellular matrix (ECM) stiffness and immune cell infiltration [45]/coupled with lysyl oxidase (LOX)–mediated collagen cross-linking [46]. LOX is an ECM remodeling enzyme that is abundantly expressed in the tumor microenvironment [47] and plays an important role in tumorigenesis and in lowering the tumor response to anticancer drugs and also confers chemoresistance [48]. Activated LOX regulates cell migration, promotes cancer malignancy [49], and is correlated with ECM stiffness and poor prognosis in breast, colorectal, head and neck, and prostate cancer [50]. Collagen deposition is the major component in ECM stiffness.

According to previous studies, COL1A1 increases cell proliferation, colony-forming efficiency, migration ability, and invasion ability [51]; besides its role in the induction of epithelial-mesenchymal transition (EMT) through the TGF-β-dependent pathway, COL1A1 might be a candidate diagnostic, prognostic, and chemoresistance biomarker for lung cancer patients [52]. Moreover, Liu et al. [6] showed that a high level of COL1A1 is indicative of a more aggressive cellular behavior and poorer prognosis in patients with breast cancer.

Inflammation is another crucial component of tumorigenesis and tumor fibrosis, which influences tumor progression and metastasis. Chronic inflammation is involved in the regulation of LOX-1 and COL1A1 expressions. TGF-β and TNF-α, pro-inflammatory cytokines that are extensively expressed in the tumor microenvironment [53], upregulate the LOX expression via the reactive oxygen species–activated NF-κB/extracellular signal-related kinase pathway, thus promoting the progression of breast cancer metastasis [54]. Furthermore, the abnormal overexpression of COL1A1 in breast cancer was accompanied by increased TGF-β1 levels [55, 56]. Our results consistent with those of Batlle and Massagué [57] showed higher levels of TGF-β and confirm its pro-oncogenic role in breast cancer. Therefore, tumor microenvironment components cooperate with inflammation and promote breast metastasis and progression. Consequently, inhibition of these components’ interaction with inflammatory cytokines may have a promising strategy to suppress breast cancer progression.

The obtained results showed that treatment with MSCs, LDR, and SeNPs either alone or combined revealed upregulation of the tumor suppressor genes (ADRP and FST) with concomitant downregulation and suppression of the oncogenic genes (Serpin, LOX-1, COL1A1, and MIF) together with inhibition of angiogenesis and activation of apoptosis. Kanapathipillai et al. [49] reported that inhibition of the LOX-1 using nanoparticles coated with a LOX inhibitory antibody binds to ECM and suppresses mammary cancer cell growth and invasion. However, Gong et al. [58] showed that high LOX expression in non-small cell lung cancer cells was associated with hypoxia-induced radioresistance. Consequently, our results showed that treatment with a single low dose of gamma radiation together with MSCs and SeNPs inhibits the expression of this enzyme. The inhibition of this enzyme was suggested as a promising therapeutic strategy for oncological diseases, including breast cancer [59].

Furthermore, Charan et al. [39] showed that the inhibition of MIF decreases breast cancer growth and metastasis via endorsing mitochondrial pathway of apoptosis and increases the levels of caspase coupled with blocking of the survival pathways. Our results work in with that in which the treatment led to downregulation of the MIF and enhanced apoptosis through increasing levels of Casp-3. Furthermore, the SeNP treatment was found to promote apoptosis in cancer cells via regulation of apoptotic proteins, such as the caspase family, p53, and ROS, thus inhibiting the malignant tumor [60, 61].

Moreover, the acidic microenvironment of malignant cells stimulates the pro-oxidant conversion of SeNPs resulting in the production of free radicals and triggering mitochondrial apoptosis via activation of caspases [23]. Consistent with the results of Janiak et al. [5] and Anzai et al. [12], LDR triggers apoptosis and/or senescence of aberrant neoplastic and cancerous cells. Furthermore, He et al. [18] showed that the combination of MSCs with radiotherapy in the treatment of breast cancer can overcome the limited curative effect and enhance the radiosensitivity of cancer cells. Moreover, it was found that the combination of MSCs with radiotherapy boosts the apoptosis of cancer cells as well as inhibits proliferation, migration, invasion, and angiogenic abilities of tumor cells [19, 62].

Additionally, Sengupta et al. [63] reported that overexpression of FST after treatment with UCMSCs induced apoptosis in breast carcinoma cells. Seachrist et al. [64] point out that FST overexpression suppresses metastatic progression of mammary carcinoma due to inhibition of the activin similarly to TGF-β. Our results coincide with the previous reports, as we detected that upregulation of ADRP and FST by different treatments either alone or combined prevents progression and metastasis of breast cancer in rats. Moreover, the treatment with MSCs, LDR, and SeNPs showed anti-inflammatory effects through hindering the levels of TGF-β and TNF-α. This may be due to the therapeutic effects of MSCs whereas they migrate towards the inflamed cancerous tissues causing suppression of tumor-promoting inflammation [65]. Interestingly, LDR stimulates the body’s immune system and immune response for the prevention and suppression of cancer metastasis [66].

The levels of angiogenesis marker VEGF were also declined after treatment confirming the effective anti-angiogenic potency of MSCs, LDR, and SeNPs either alone or combined. Modulation of the VEGF by SeNPs hindered the angiogenic signaling in cancer cells, therefore, impairing the proliferation and the growth signaling in the tumor microenvironment [23]. Previous results reported that MSCs reduced the expression of VEGF in breast cancer cells, causing inhibition of angiogenesis [67]. Additionally, it was found that treatment with nano-Se or radiation alone inhibits cell proliferation, migration, and invasion, as well as inducing apoptosis. However, their combination has a synergistic effect, which is more obvious due to the promotion of each other [22].

## Conclusion

Overall, this study confirms the therapeutic efficacy of low-dose radiation and the tumor-targeting characteristic of MSCs combined with SeNPs against breast cancer. MSCs, SeNPs, and LDR notably modulated the expression of multiple tumor suppressors and promoter genes playing a role in breast cancer induction and suppression. Furthermore, the antitumor effect shown here is due to induction of apoptosis as well as disruption neovascularization, suppression of tumor-promoting inflammation, and the growth signaling in tumor microenvironment thereby decreasing tumor cell proliferation, thus offering a promising and effective treatment option for advanced breast cancer, but further studies are still needed to determine the exact mechanism.

## Data Availability

All data are presented in the manuscript.

## Abbreviations

ADRP: Adipocyte differentiation–related protein
BM-MSCs: Bone marrow–derived mesenchymal stem cells
CA15-3: Carcinoma antigen
caspase-3: Cysteine-aspartic acid protease-3
COL1A1: Collagen type I alpha 1
FST: Follistatin
LOX-1: Lysyl oxidase–like 1
MIF: Macrophage migration inhibitory factor
SeNPs: Selenium nanoparticles
LDR: Low dose of gamma radiation
SerpinE: Serpin peptide inhibitor clade E
TGF-β: Transforming growth factor-beta
TNF-α: Tumor necrosis factor-α
VEGF: Vascular endothelial growth factor
